# New therapeutic strategy for atopic dermatitis by targeting CHI3L1/ITGA5 axis

**DOI:** 10.1002/ctm2.739

**Published:** 2022-02-20

**Authors:** Yong Sun Lee, Ji Eun Yu, Min Ji Kim, Hyeon Joo Ham, Seong Hee Jeon, Jaesuk Yun, Suk‐Gil Song, Chong‐Kil Lee, Sang Bae Han, Dong Ju Son, Jin Tae Hong

**Affiliations:** ^1^ College of Pharmacy and Medical Research Center Chungbuk National University Cheongju Republic of Korea

Dear Editor,

Atopic dermatitis (AD) is a chronic recurrent inflammatory skin disease that is difficult to treat despite the discovery of various disease targets and development of therapeutics. Previous clinical studies have shown that chitinase 3‐like protein 1 (CHI3L1), also known as YKL‐40, is associated with the onset and severity of AD.^1^, ^2^, ^3^ Recently, CHI3L1 has attracted attention as a new therapeutic target for treating various diseases, including cancer and autoimmune diseases.^4^ Our gene‐disease network analysis also showed CHI3L1 is associated with various diseases, including inflammatory diseases (Figure S1). However, the role of CHI3L1 in AD pathogenesis is not well‐understood. Previously, we provided the first direct evidence that inhibition of CHI3L1 by K284‐6111, a novel CHI3L1 inhibitor, ameliorates AD‐like skin inflammation.^5^ Here, we further investigated the role of CHI3L1 in AD pathogenesis and its underlying mechanism using CHI3L1 knockout (KO) mice and a CHI3L1‐blocking antibody (CHI3L1‐Ab), and based on outcomes, propose that a CHI3L1‐targeted therapeutic strategy can be used to treat AD.

To determine if CHI3L1 plays a role in AD pathogenesis, CH3L1‐KO and wild‐type (WT) mice were treated with phthalic anhydride (PA) to induce AD‐like skin inflammation. WT mice displayed elevated CHI3L1 levels in the epidermal/dermal layers, particularly in epidermal keratinocytes, and blood serum, as well as typical AD‐like symptoms such as erythema, edema, hyperkeratosis and epidermal thickening. In comparison, CHI3L1‐KO mice showed significantly reduced symptoms and abrogated CHI3L1 levels (Figure 1). In addition, PA‐induced pro‐inflammatory marker protein (cyclooxygenase‐2 and inducible nitric oxide synthase) expression, inflammatory immune cell infiltration, inflammatory cytokine and chemokine (interleukin [IL]‐1β, IL‐4, IL‐6, IL‐13, thymic stromal lymphoprotein [TSLP] and C‐C motif chemokine 22 [CCL22]) and IgE and histamine levels were decreased in the skin tissue or serum compared to those in WT mice (Figure S2). Moreover, expression of genes encoding inflammatory cytokines and chemokines induced by treatment with a mixture of tumour necrosis factor (TNF)‐α and interferon (IFN)‐γ, an inflammatory stimulant that induces AD‐like features, was inhibited by CHI3L1 knockdown, whereas it was up‐regulated by CHI3L1 overexpression in HaCaT cells (Figure S3). Taken together, these findings indicate that CHI3L1 greatly contributes to the AD pathogenesis.

**FIGURE 1:**
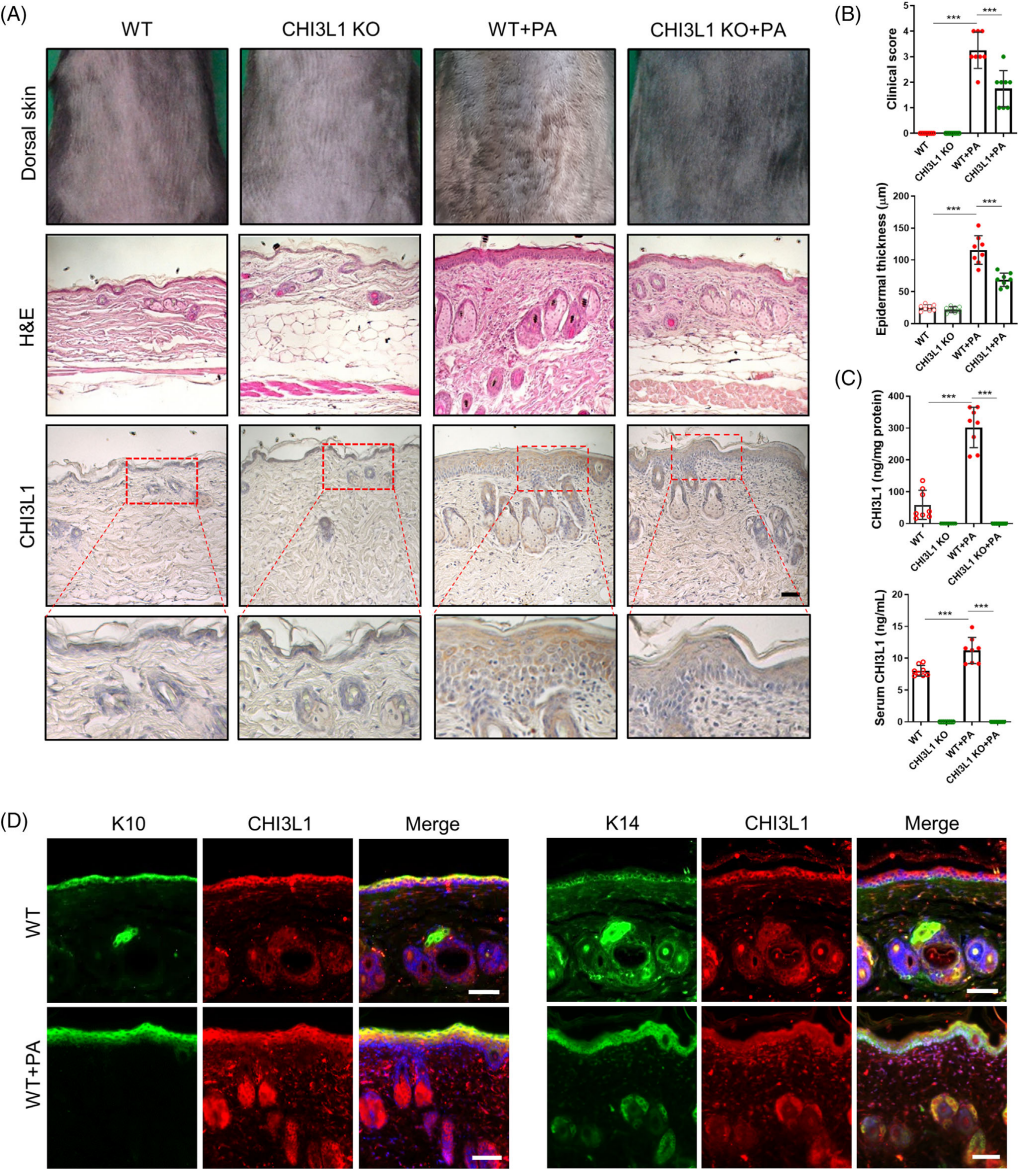
Chitinase 3‐like protein 1 (CHI3L1) knockout (KO) inhibits atopic dermatitis (AD) development. Wild‐type (WT) and CHI3L1 KO mice were treated with 5% phthalic anhydride (PA) for 4 weeks. (A) Morphological (upper images) and histological changes (middle images) in mice after 4‐week treatment. Expression of CHI3L1 in PA‐induced skin tissues by immunohistochemistry analysis (bottom images). The photographs are representative of each group of mice. Scale bar, 50 μm. (B) Bar graphs indicate clinical score (upper panel) and epidermal thickness (lower panel). *n *= 8. (C) The concentration of CHI3L1 in mouse skin tissues (upper panel) and serum (lower panel). *n *= 8. (D) Representative immunofluorescence images showing CHI3L1, cytokeratin 10 (K10) and cytokeratin 14 (K14) stained cells in skin tissues. Scale bar, 50 μm. Data are expressed as the mean ± standard deviation (SD). ***p < .001

To explore the underlying mechanism of CHI3L1 in AD pathogenesis, we performed gene‐network analysis using Humanbase, an interactive platform of data‐driven predictions of gene regulation and interaction, which showed that CHI3L1 is linked to numerous genes of interest, including integrin beta2 (ITGB2) (Figure 2A). Integrins are transmembrane adhesion receptors with important roles in biological and pathological processes.^6^ Previous studies have shown that integrin alpha (ITGA) is overexpressed in atopic skin,^7^ and CHI3L1‐mediated biological and pathological effects are through interactions with integrins.^8^ To determine the association between CHI3L1 and integrins including ITGB2, ITGA2, ITGA5 and ITGA6, we compared the mRNA expression levels of these integrins in the skin of the PA‐induced AD model. Expression of all tested integrins increased in WT mice but decreased in CHI3L1‐KO mice; particularly, ITGA5 expressions were significantly down‐regulated by CHI3L1‐KO (Figure 2B,C). Additionally, gene silencing and overexpression experiments in HaCaT cells demonstrated that ITGA5 expression was more affected by CHI3L1 compared to ITGA6 (Figure 2D,E). Differences in protein expression of ITGA5 were further confirmed in TNF‐α/IFN‐γ‐treated HaCaT cells (Figure 2F) and skin tissues (Figure 2G). We further analysed the gene expression levels of pro‐inflammatory mediators after knocking down ITGA5 expression in TNF‐α/IFN‐γ‐treated HaCaT cells and found that the mRNA expressions of IL‐1β, IL‐6, TSLP and CCL22 were significantly decreased (Figure 2H).

**FIGURE 2:**
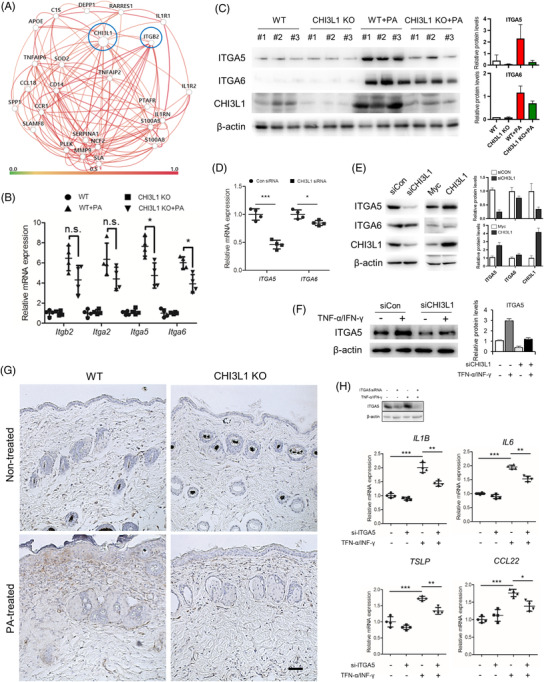
Chitinase 3‐like protein 1 (CHI3L1) regulates ITGA5 expression. (A) The gene‐network map of CHI3L1 and its predicted gene interactions. (B) mRNA expression of *Itgb2*, *Itga2*, *Itga5* and *Itga6* in animal model skin tissues determined by quantitative PCR (qPCR). *n *= 4. (C) Expression of ITGA5, ITGA6 and CHI3L1 in phthalic anhydride (PA)‐induced skin tissues detected by Western blot analysis. *n *= 3. (D) HaCaT cells were transfected with human CHI3L1 siRNA (20 nM) for 24 h. mRNA expression of *ITGA5* and *ITGA6* was determined using qPCR. *n *= 4. (E) Expression of ITGA5 and ITGA6 in CHI3L1 knockdown or CHI3L1‐overexpressing HaCaT cells detected by Western blot analysis. (F) HaCaT cells were transfected with CHI3L1 siRNA (20 nM). After 24 h, the cells were treated with tumour necrosis factor (TNF)‐α and interferon (IFN)‐γ (20 ng/ml) for 4 h. Expression of ITGA5 was analysed by Western blot analysis. (G) Expression of ITGA5 in PA‐induced skin tissues detected using immunohistochemistry. Scale bar, 50 μm. (H) HaCaT cells were transfected with human ITGA5 siRNA (20 nM). After 24 h, the cells were treated with tumour necrosis factor (TNF)‐α and IFN‐γ (20 ng/ml) for 4 h. Knockdown efficiency was determined by Western blot analysis, and mRNA expression of interleukin (IL)1B, IL6, thymic stromal lymphoprotein (TSLP) and *CCL22* was determined using qPCR. *n *= 4. Data are expressed as the mean ± SD. *p < .05, **p < .01 and ***p < .001

Notably, transcription factor prediction analysis showed that ITGA5 contains 13 putative nuclear factor (NF)‐κB binding sites (Figure S4A). Interestingly, ITGA5 expression was inhibited by not only NF‐κB p65 siRNA in TNF‐α/IFN‐γ‐treated HaCaT cells (Figure S4B), but also in CHI3L1‐overexpressing cells treated with an NF‐κB inhibitor (Figure S4C), indicating that NF‐κB is a key transcription factor regulating ITGA5 expression. As NF‐κB is a critical regulator in AD,^9^ and CHI3L1 is known to bind to the RAGE (receptor for advanced glycation end‐products) receptor to contribute to NF‐κB activation, we investigated whether CHI3L1 modulates NF‐κB signaling.^10^ CHI3L1 overexpression promoted NF‐κB signaling activation in resting and TNF‐α/IFN‐γ‐treated HaCaT cells (Figure S5A,B). In contrast, CHI3L1 knockdown reduced both TNF‐α/IFN‐γ‐induced phosphorylation of IκBα and nuclear translocation of p50/p65 (Figure S5C,D). In addition, CHI3L1 knockdown together with the NF‐κB inhibitor synergistically inhibited IL‐1β, IL‐6, TSLP and CCL22 production (Figure S5E). Notably, PA‐induced NF‐κB activation in the skin tissues decreased in CHI3L1‐KO mice compared to in WT mice (Figure S5F,G). These results suggest that CHI3L1 induces inflammatory responses by activating NF‐κB signaling, which may lead to AD‐related skin inflammation. Collectively, our findings suggest that CHI3L1‐related AD pathogenesis is associated with the regulation of the NF‐κB/ITGA5 axis.

After confirming that CHI3L1 regulates AD‐related skin inflammation, we examined the therapeutic effect of CHI3L1‐antibody (Ab). Administration of a CHI3L1‐Ab alleviated PA‐induced AD development and the inflammatory response and suppressed the production of CHI3L1 and ITGA5 and activation of NF‐κB (Figure 3A–D). Furthermore, the analysis of the reconstructed human skin (RHS) tissue AD model confirmed that CHI3L1‐Ab treatment reduced skin inflammation by inhibiting the CHI3L1/NF‐κB/ITGA5 axis (Figure 3E–I). Additionally, we demonstrated that ITGA5‐Ab treatment reduced epidermal thickness in the same RHS AD model (Figure S6).

**FIGURE 3:**
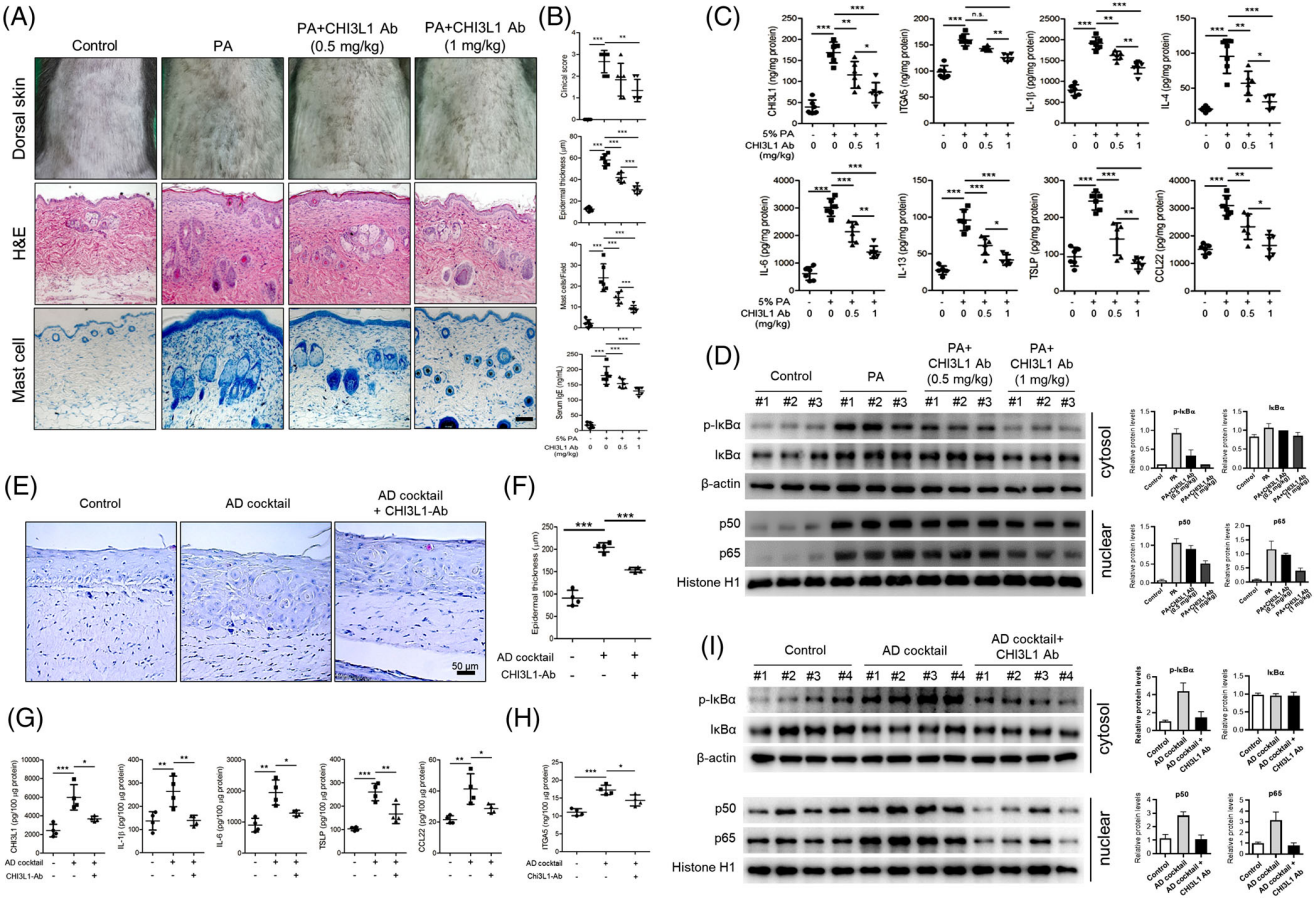
Blockade of chitinase 3‐like protein 1 (CHI3L1) suppresses atopic dermatitis (AD) skin inflammation in phthalic anhydride (PA)‐induced mouse model and AD‐like reconstructed human skin (RHS) model. (A–D) Wild‐type (WT) mice were treated with 5% PA for 4 weeks. From the third week, CHI3L1 antibody (CHI3L1 Ab; .5 and 1 mg/kg per mouse) was intravenously injected via the tail vein at 3 h after PA treatment. (A) Morphologic (upper images) and histologic changes (middle images) in the mice after CHI3L1 antibody therapy. Scale bar, 50 μm. (B) Bar graphs indicate the clinical score, epidermal thickness, mast cell number and serum IgE levels. *n* = 6. (C) Protein levels of CHI3L1, ITGA5, interleukin (IL)‐1β, IL‐4, IL‐6, IL‐13, thymic stromal lymphoprotein (TSLP) and CCL22 in PA‐induced skin tissues. *n *= 6. (D) Expression of phosphorylated IκBα in cytosolic fractions and nuclear translocation of p50 and p65 in nuclear fractions in PA‐induced skin tissues by Western blot analysis. *n *= 3. (E–I) RHS model cells were cultured with AD cocktail with/without CHI3L1 Ab (500 ng/ml) for 6 days. (E) Histological changes in AD‐RHS model. Scale bar, 50 μm. (F) Bar graph presents epidermal thickness. *n* = 4. (G) Protein levels of CHI3L1, IL‐1β, IL‐6, TSLP and CCL22 in AD‐RHS model. *n* = 4. (H) Levels of ITGA5 in AD‐RHS model. *n *= 4. (I) Expression of phosphorylated IκBα in cytosolic fractions and nuclear translocation of p50 and p65 in nuclear fractions in AD‐RHS model determined by Western blot analysis. *n *= 3. Data are expressed as the mean ± SD. *p < .05, **p < .01 and ***p < .001

Finally, we found that the serum levels of CHI3L1, ITGA5 and other AD biomarkers were significantly increased in sera from patients with AD (Figure 4A,B [left] and Figure S7A). Receiver operating characteristic curve analysis indicated that CHI3L1 and ITGA5 are more suitable diagnostic markers for AD compared to other biomarkers (Figure 4A,B [right] and Figure S7B). Additionally, Spearman's correlation analysis showed that serum level of CHI3L1 significantly correlated with the serum level of ITGA5 (Figure 4C). These results suggest that CHI3L1 and ITGA5 are potent diagnostic and prognostic biomarkers for AD.

**FIGURE 4:**
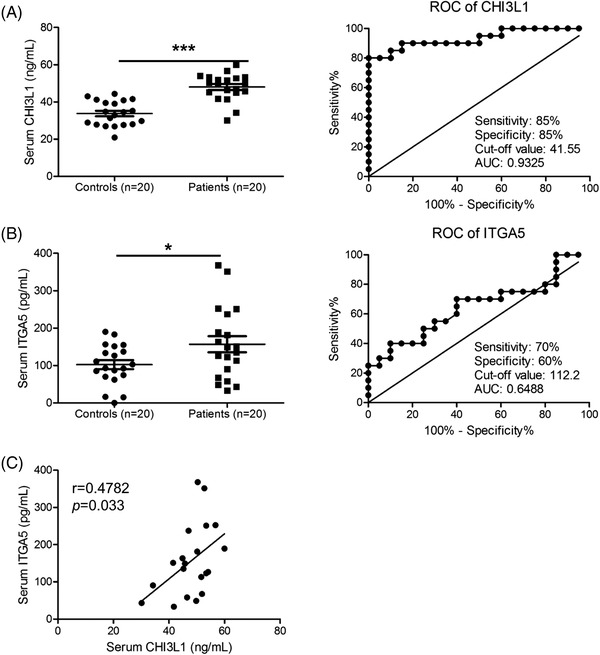
Clinical relationship between Chi3L1 and ITGA5 in patients with atopic dermatitis (AD). (A and B) Serum levels and receiver operating characteristic curve of chitinase 3‐like protein 1 (CHI3L1) (A) and ITGA5 (B) in patients with AD and healthy controls. *n* = 20. (C) Correlation between CHI3L1 and ITGA5. *R* value indicates Spearman's correlation co‐efficient. Data are expressed as the mean ± SD. *p < .05, and ***p < .001

In conclusion, CHI3L1 mediates AD‐like skin inflammation by regulating NF‐κB‐dependent ITGA5 expression. Furthermore, the inhibition of CHI3L1 by CHI3L1‐Ab suppresses AD‐like symptoms and inflammatory responses by regulating CHI3L1, the NF‐κB/ITGA5 axis. Our findings provide experimental rationale for developing therapeutic strategies for AD by targeting CHI3L1.

## CONFLICT OF INTEREST

The authors declare that they have no competing interests.

## Supporting information

SUPPORTING INFORMATION
