# Supplementary material for: New therapeutic strategy for atopic dermatitis by targeting CHI3L1/ITGA5 axis
Source: Clin Transl Med. 2022 Feb 20;12(2):e739. doi: 10.1002/ctm2.739 (PMC8858621; doi:10.1002/ctm2.739)
Supplement: Supplementary file 1 — SUPPORTING INFORMATION [file CTM2-12-e739-s001.docx]

**Supporting Information**

**New therapeutic strategy for atopic dermatitis by targeting CHI3L1/ITGA5 axis**

Yong Sun Lee^*^, Ji Eun Yu^*^, Min Ji Kim, Hyeon Joo Ham, Seong Hee Jeon, Jaesuk Yun, Suk-Gil Song, Chong-Kil Lee, Sang Bae Han^#^ , Dong Ju Son^#^ and Jin Tae Hong^#^

College of Pharmacy and Medical Research Center, Chungbuk National University, Osongsaengmyeong 1-ro 194-21, Osong-eup, Heungduk-gu, Cheongju, Chungbuk, 28160, Republic of Korea

* These authors contributed equally to this work.

^#^ **Correspondences:** Drs. Jin Tae Hong (jinthong@chungbuk.ac.kr), Dong Ju Son (sondj1@chungbuk.ac.kr) and Sang Bae Han (shan@chungbuk.ac.kr), College of Pharmacy and Medical Research Center, Chungbuk National University, Osongsaengmyeong 1-ro, Osong-eup, Heungdeok-gu, Cheongiu, Chungbuk, 28160, Republic of Korea, Tel: +82-42-261-2813, Fax: +82-42-268-2732

**Supplemental Materials and Methods**

**Animals and ethical approval**

The experimental protocols were carried out according to the guidelines for animal experimentation of the Institutional Animal Care and Use Committee of the Laboratory Animal Research Center at Chungbuk National University, Korea (ethical approval number: CBNUA-1206-18-01). All efforts were made to minimize animal suffering and reduce the number of animals used. All mice were housed with three mice per cage with automatic temperature control (21–25°C), relative humidity (45–65%), and 12-h light-dark cycle illumination, and were provided filtered tap water and rodent chow diet *ad libitum* throughout the experiment. Wild-type (WT) (C57BL/6) mice were purchased from DBL (Eumsung, Korea). CHI3L1 knockout (KO) mice were generated as described in a previous study.[^1^](#_ENREF_1)

**PA-induced AD model**

Age-matched male WT mice and CHI3L1 KO mice (8-week old) in the C57BL/6 background strain were used. To induce atopic dermatitis (AD) on the skin, 100 μL of 5% phthalic anhydride (PA) solution or vehicle (acetone: olive oil in a 4:1, *v*/*v*) was spread on the dorsum of the back skin three times per week for 4 weeks. Clinical scores were evaluated as none (0), mild (1), moderate (2), or severe (3) according to the AD symptoms, erythema (redness), scaling, and itching. For the CHI3L1 therapy experiment, anti-CHI3L1 antibody (0.5 or 1 mg/kg per mouse; polyclonal, Cat# ab180569, Abcam, Cambridge, UK) was intravenously injected via the tail vein at 3 h after PA treatment in the third week.

**AD-like reconstructed human skin model**

A reconstructed human skin (RHS) model was purchased from TEGO Science, Inc. (Neoderm®-ED, Seoul, Korea). The RHS model contains the epidermis and dermis and generates AD-like inflammation when an inflammatory cocktail is applied, as previously described, with some modifications [^2^](#_ENREF_2)^,^[^3^](#_ENREF_3). Briefly, an inflammatory cocktail (AD cocktail; IL-4: 30 ng/mL; IL-13: 30 ng/mL; TNF-α: 3.5 ng/mL; PeproTech, Inc., Rocky Hill, NJ, USA) was added with or without CHI3L1 antibody (500 ng/mL; Abcam) to the maintenance medium (TEGO Science) for 6 days. The culture medium was changed every 48 h. The culture medium was supplied by TEGO Science Inc.

**Western blot analysis**

Western blot analysis was performed as previously described.[^4^](#_ENREF_4) Total 10 μg of proteins were subjected to SDS-PAGE for separation and then transferred to PVDF membrane. The membranes were incubated with the following specific primary antibodies: β-actin (Santa Cruz Biotechnology, Dallas, TX, USA); iNOS, COX-2, CHI3L1, ITGA5, and ITGA6 (Abcam), and p50, p65, p-lκBα, and IκBα (Cell Signaling Technology, Danvers, MA, USA). Information on the antibodies is presented in Table S1.

**Immunohistochemistry**

Back skins were removed from the mice, fixed with 10% formalin, embedded in paraffin wax, routinely processed, and then sectioned into 10-μm-thick slices. Hematoxylin and eosin staining and immunohistochemistry were performed as previously described.[^4^](#_ENREF_4) Hematoxylin and eosin stained sections were viewed under a light microscope (Olympus, Tokyo, Japan). Toluidine blue was used to stain the mast cells. For immunohistochemistry, the skin tissue sections were blocked with 3% normal goat serum diluted in PBS, for 30 min; the sections were then incubated with antibodies for phospho-p65, CHI3L1, and ITGA5 (Abcam) and F4/80 (Cell Signaling Technology), at the appropriate dilution in blocking serum, for overnight at 4 ̊C. The slides were washed in PBS, followed by the avidin–biotin–peroxidase complex (#PK-6101, Vector Laboratories, Burlingame, CA). The slides were washed, and the peroxidase reaction was developed with diaminobenzidine and peroxide (#SK-4100, Vector Laboratories), mounted in Aqua-Mount, and evaluated under a light microscope (Olympus).

**Immunofluorescence**

Back skins were removed from the mice, fixed with 10% formalin, embedded in paraffin wax, routinely processed, and then sectioned into 10-μm-thick slices. Primary antibodies were incubated for overnight at 4 °C and Alexa Fluor 488 (#A32723, #A32731, Invitrogen) or Texas Red (#T-862, #T-2767, Invitrogen) conjugated secondary antibodies were incubated for 1h at room temperature. Fixed cells were incubated with 1 μg/ml of DAPI (#**D9542, Sigma-Aldrich**) for 5 min at room temperature and then covered with Fluoromount-G Mounting Medium (#0100-01, Southern Biotech, Birmingham, AL). Cells were visualized using Ziess AxioObserver (Carl Zeiss, Oberkochen, Germany) fluorescence microscope system. Digital images were analyzed using the imageJ (NIH) or ZEN 2.1 (Carl Zeiss) software. The primary antibodies were as follows: CHI3L1 and K10 (Abcam), K10 (Invitrogen, Carlsbad, CA), and K14 (Santa Cruz Biotechnology). Information on the antibodies is shown in Table S1.

**Quantitative real-time PCR**

Total RNA was extracted from the skin tissues or cells using the Ribo^EX^ RNA Extraction Kit (GeneAll Biotechnology, Seoul, Korea), and cDNA was synthesized using a HighCapacity RNA-to-cDNA kit (Applied Biosystems, Foster City, CA, USA). Quantitative real-time PCR was performed using specific primers with SYBR Green master mix (ELPIS Biotech, Daejeon, Korea) in a StepOnePlus^TM^ PCR System (Applied Biosystems) (Table S2). The thermocycling conditions were as follows: initial denaturation of 3 min at 94.0°C, followed by 40 cycles of 94.0°C for 1 min, 58.0°C for 30 s, and 72.0°C for 30 s according to the manufacturer’s instructions. The values obtained for target gene expression were normalized to GAPDH or 18S and quantified relative to the expression in control samples.

**Enzyme-linked immunosorbent assay**

Blood samples were collected from the mice at the end of the study period. Mouse cytokines (IL-1β, IL-4, IL-6, IL-13, TSLP, and CCL22), ITGA5, and serum lgE levels were measured using enzyme-linked immunosorbent assay (ELISA) kits provided by KOMA Biotech (Seoul, Korea), MyBioSource (SanDiego, CA, USA), and R&D Systems (Minneapolis, MN, USA) according to the manufacturer’s instructions. Mouse tissue and serum CHI3L1 and human serum CHI3L1 levels were analyzed using specific ELISA kits purchased from R&D Systems according to the manufacturer’s instructions. Human cytokines (IL-1β, IL-4, IL-6, IL-13, TSLP, and CCL22) and human ITGA5 levels were measured using ELISA kits provided by KOMA Biotech, Invitrogen (Carlsbad, CA, USA), and R&D Systems (Minneapolis, MN, USA) according to the manufacturer’s instructions. Detailed information is provided in Supplementary Table S1.

**Cell culture and transfection**

HaCaT cells were kindly provided by Dr. Do Young Yoon (Konkuk University, Seoul, Korea). HaCaT cells were grown at 37°C in Dulbecco’s Modified Eagle Medium supplemented with 10% fetal bovine serum, 100 U/mL penicillin, and 100 μg/mL streptomycin in a humidified atmosphere of 5% CO_2_. HaCaT cells were transfected with human CHI3L1 siRNA, human ITGA5 siRNA (Origene Technologies, Inc., Rockville, MD, USA), human p65 siRNA (Santa Cruz Biotechnology), and its control siRNAs using Lipofectamine RNAiMAX reagent (Invitrogen), and then stimulated with TNF-α/IFN-γ (20 ng/mL; Peprotech) for the times indicated in the figure legends. To overexpress CHI3L1, HaCaT cells were transfected with the pcDNA3.1(+)-6xMyc-CHI3L1 vector or control vector using Lipofectamine 3000 transfection reagent (Invitrogen).

**Luciferase assay**

HaCaT cells were transfected with a luciferase-expressing NF-κB plasmid vector (Promega, Madison, WI, USA) for 24 h. The cells were then transfected with human CHI3L1 siRNA for another 24 h. The transfected cells were treated with TNF-α/lFN-γ (20 ng/mL) for 24 h. Luciferase activity was measured using a luciferase assay kit (GeneCopoeia, Rockville, MD, USA) following the manufacturer’s instructions.

**Human samples**

Human serum samples from patients with AD and normal controls were obtained from the Chungbuk National University Hospital Biobank and the Korea Institute of Radiological & Medical Sciences Radiation Biobank. All studies using human samples were conducted in accordance with the Declaration of Helsinki and were approved by the Ethics Committee of Chungbuk National University Medical Center (IRB No. CBNU-201902-BR-786-01).

**Web-based analysis**

The gene-disease network of CHI3L1 was analyzed using the Open Targets platform (https://www.opentargets.org/) and Disease-Connect web server (<http://www.disease-connect.org>). The Open Targets platform is based on rare and common disease genetics, somatic mutations in cancer, transcriptomics, approved drugs and clinical candidates, animal models, biochemical pathways, and text mining from the medical literature. The Disease-Connect web server is based on various sources, genome-wide association studies, the Online Mendelian Inheritance in Man, GeneRIF, and GeneWays. The CHI3L1-related gene network was analyzed using Humanbase (biological process-specific interactions set as global; https://hb.flatironinstitute.org/). Humanbase provides data-driven predictions of gene expression, function, regulation, and interactions in humans, particularly in the context of specific cell types/tissues and human diseases.

**Statistical analysis**

The experiments were conducted in triplicate, and all experiments were repeated at least three times and showed similar results. All statistical analyses were performed using GraphPad Prism 4 software (GraphPad, Inc., San Diego, CA, USA). Group differences were analyzed using one-way analysis of variance followed by Tukey's multiple comparison test. All values are presented as the mean ± standard deviation. Significance was set at *p* < 0.05.

**References**

1. Kim KC, Yun J, Son DJ, et al. Suppression of metastasis through inhibition of chitinase 3-like 1 expression by miR-125a-3p-mediated up-regulation of USF1. *Theranostics*. 2018;8(16):4409-4428. doi:10.7150/thno.26467

2. Danso MO, van Drongelen V, Mulder A, et al. TNF-α and Th2 Cytokines Induce Atopic Dermatitis–Like Features on Epidermal Differentiation Proteins and Stratum Corneum Lipids in Human Skin Equivalents. *Journal of Investigative Dermatology*. 2014/07/01/ 2014;134(7):1941-1950. doi:<https://doi.org/10.1038/jid.2014.83>

3. Lee YS, Han S-B, Ham HJ, et al. IL-32γ suppressed atopic dermatitis through inhibition of miR-205 expression via inactivation of nuclear factor-kappa B. *Journal of Allergy and Clinical Immunology*. 2020/07/01/ 2020;146(1):156-168. doi:<https://doi.org/10.1016/j.jaci.2019.12.905>

4. Lee YS, Lee CH, Bae JT, et al. Inhibition of skin carcinogenesis by suppression of NF-κB dependent ITGAV and TIMP-1 expression in IL-32γ overexpressed condition. journal article. *Journal of Experimental & Clinical Cancer Research*. November 28 2018;37(1):293. doi:10.1186/s13046-018-0943-8

**Supplementary Tables**

Table S1. Information of antibodies, kits, and reagents

| **Name** | **Cat. No.** | **Application** | **Supplier** |
| --- | --- | --- | --- |
| **Antibody** | | | |
| CHI3L1 | ab180569 | WB, IHC-P | Abcam |
| ITGA5 [EPR7854] | ab150361 | WB, IHC-P | Abcam |
| ITGA6 | A3236 | WB | Abclonal |
| iNOS | ab15323 | WB | Abcam |
| COX-2 [EPR12012] | ab179800 | WB | Abcam |
| Phospho-p65 | ab86299 | IHC-P | Abcam |
| Histone H1 | ab61177 | WB | Abcam |
| Phospho-IκBα | 5209 | WB | Cell Signaling |
| IκBα | 4814 | WB | Cell Signaling |
| p65 (D14E12) | 8242 | WB | Cell Signaling |
| F4/80 (D2S9R) | 70076 | IHC-P | Cell Signaling |
| Phospho-ERK (D13.14.4E) | 4370 | WB | Cell Signaling |
| ERK (137F5) | 4695 | WB | Cell Signaling |
| Myc-tag (71D10) | 2278 | WB | Cell Signaling |
| p50 (NLS) | sc-114 | WB | Santa Cruz Bio |
| Cytokeratin 10 | MA1-06319 | IF | Invitrogen |
| Cytokeratin 14 (LL001) | Sc-53253 | IF | Santa Cruz Bio |
| β-actin (C4) | sc-47778 | WB | Santa Cruz Bio |
| **Kits** | | | |
| Human Chi3L1 ELISA Kit | DY2599 | ELISA | R&D systems |
| Human ITGA5 ELISA Kit | DY1864-05 | ELISA | R&D systems |
| Human CCL22 ELISA Kit | DY336 | ELISA | R&D systems |
| Human IL-4 ELISA Kit | BMS225-2 | ELISA | Invitrogen |
| Human IL-13 ELISA Kit | BMS231-3 | ELISA | Invitrogen |
| Human IL-1β ELISA Kit | K0331800HS | ELISA | KOMA Biotech |
| Human IL-6 ELISA Kit | K0331194HS | ELISA | KOMA Biotech |
| Mouse Chi3L1 ELISA Kit | DY2649 | ELISA | R&D systems |
| Mouse ITGA5 ELISA Kit | MBS2887565 | ELISA | MyBioSource |
| Mouse IL-1β ELISA Kit | K0331231 | ELISA | KOMA Biotech |
| Mouse IL-4 ELISA Kit | K0331144 | ELISA | KOMA Biotech |
| Mouse IL-6 ELISA Kit | K0331230 | ELISA | KOMA Biotech |
| Mouse IL-13 ELISA Kit | K0331201 | ELISA | KOMA Biotech |
| Mouse IgE ELISA Kit | K3231082 | ELISA | KOMA Biotech |
| Mouse TSLP ELISA Kit | MTLP00 | ELISA | R&D systems |
| Mouse CCL22 ELISA Kit | MCC220 | ELISA | R&D systems |
| qPCR 2x Master Mix (SYBR Green) | EBT-1802 | qPCR | ELPIS Biotech |
| High-Capacity cDNA Reverse Transcription Kit | 4368813 | qPCR | Applied Biosystems |
| VECTASTAIN® Elite ABC-HRP Kit | PK-6101 | IHC-P | Vector Laboratories |
| NovaUltra Toluidine Blue Stain Kit | IW-3013 | IHC-P | IHC World |
| Secrete-Pair^TM^ Dual Luciferase Assay Kit | LF033 | Luciferase assay | GeneCopoeia |
| **Reagents** | | | |
| Recombinant human TNF-α | 300-01A |  | PeproTech |
| Recombinant human IFN-γ | 300-02 |  | PeproTech |
| Recombinant human IL-4 | 200-04 |  | PeproTech |
| Recombinant human IL-13 | 200-13 |  | PeproTech |
| Lipofectamine® 3000 | L3000015 | Transfection | Invitrogen |
| Lipofectamine® RNAiMAX | 13778150 | Transfection | Invitrogen |
| Phthalic anhydride | 320064 |  | Sigma Aldrich |
| BAY 11-7082 (NF-κB inhibitor) | B5556 |  | Sigma |

Table S2. Primer sequence information

| **Primer** | **Species** | **Sequence (Forward)** | **Sequence (Reverse)** |
| --- | --- | --- | --- |
| **qPCR** | | | |
| *18S* |  | AGGAATTGACGGAAGGGCACCA | GTGCAGCCCCGGACATCTAAG |
| *Il1b* | mouse | CCTTCCAGGATGAGGACATGA | TGAGTCACAGAGGATGGGCTC |
| *Il4* | mouse | GGTCTCAACCCCCAGCTAGT | GCCGATGATCTCTCTCAAGTGAT |
| *Il6* | mouse | GAGGATACCACTCCCAACAGACC | AAGTGCATCATCGTTGTTCATACA |
| *Il13* | mouse | CCTGGCTCTTGCTTGCCTT | GGTCTTGTGTGATGTTGCTCA |
| *Tslp* | mouse | ACGGATGGGGCTAACTTACAA | AGTCCTCGATTTGCTCGAACT |
| *Ccl22* | mouse | CTCTGCCATCACGTTTAGTGAA | GACGGTTATCAAAACAACGCC |
| *Itgb2* | mouse | CAGGAATGCACCAAGTACAAAGT | CCTGGTCCAGTGAAGTTCAGC |
| *Itga2* | mouse | TGTCTGGCGTATAATGTTGGC | CTTGTGGGTTCGTAAGCTGCT |
| *Itga5* | mouse | CTTCTCCGTGGAGTTTTACCG | GCTGTCAAATTGAATGGTGGTG |
| *Itga6* | mouse | TGCAGAGGGCGAACAGAAC | GCACACGTCACCACTTTGC |
| *GAPDH* | human | GACAAGCTTCCCGTTCTCAG | CAATGACCCCTTCATTGACC |
| *IL1B* | human | ATGATGGCTTATTACAGTGGCAA | GTCGGAGATTCGTAGCTGGA |
| *IL6* | human | ACTCACCTCTTCAGAACGAATTG | CCATCTTTGGAAGGTTCAGGTTG |
| *TSLP* | human | TAGCAATCGGCCACATTGCC | CTGAGTTTCCGAATAGCCTG |
| *CCL22* | human | GTTGTCCTCGTCCTCCTTGC | GGAGTCTGAGGTCCAGTAGAAGTG |
| *ITGA5* | human | GGCTTCAACTTAGACGCGGAG | TGGCTGGTATTAGCCTTGGGT |
| *ITGA6* | human | GGCGGTGTTATGTCCTGAGTC | AATCGCCCATCACAAAAGCTC |
| **Genotyping** | | | |
| *Chi3l1* | WT | TGACACTGGATGGCACAACT | TGCACAGGAAGGTTGGAT |
| *Chi3l1* | KO | CTTACCAGACGCCATCCTTCGT | CATGTTACCATCCCAACACAGC |

**Supplementary Figures**


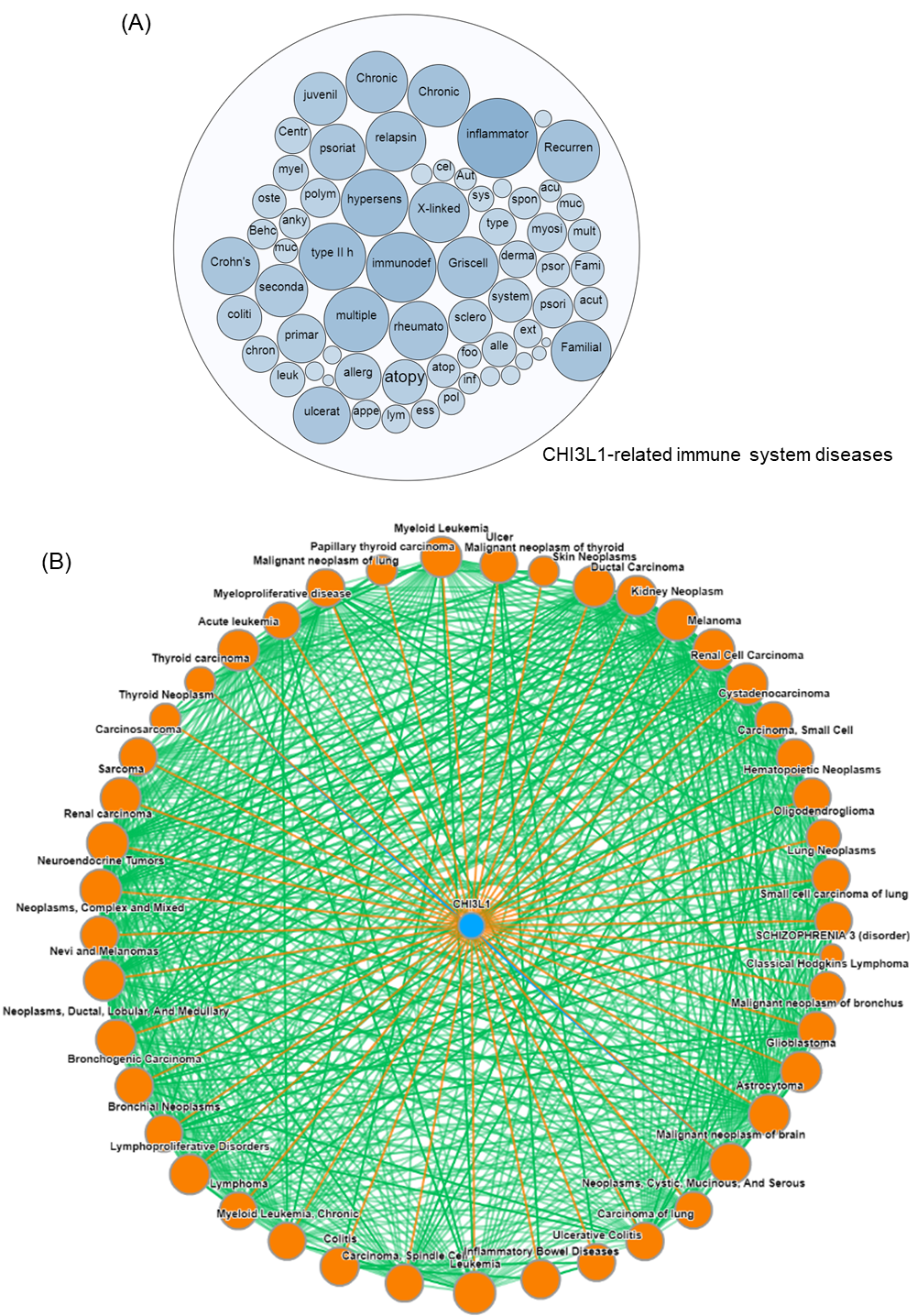


**Supplementary Figure S1. CHI3L1 is associated with various diseases, including inflammatory diseases.** (A and B) CHI3L1-disease association was analyzed using Open Targets platform (A; Bubble map) and Disease-Connect web server (B; network map).

**
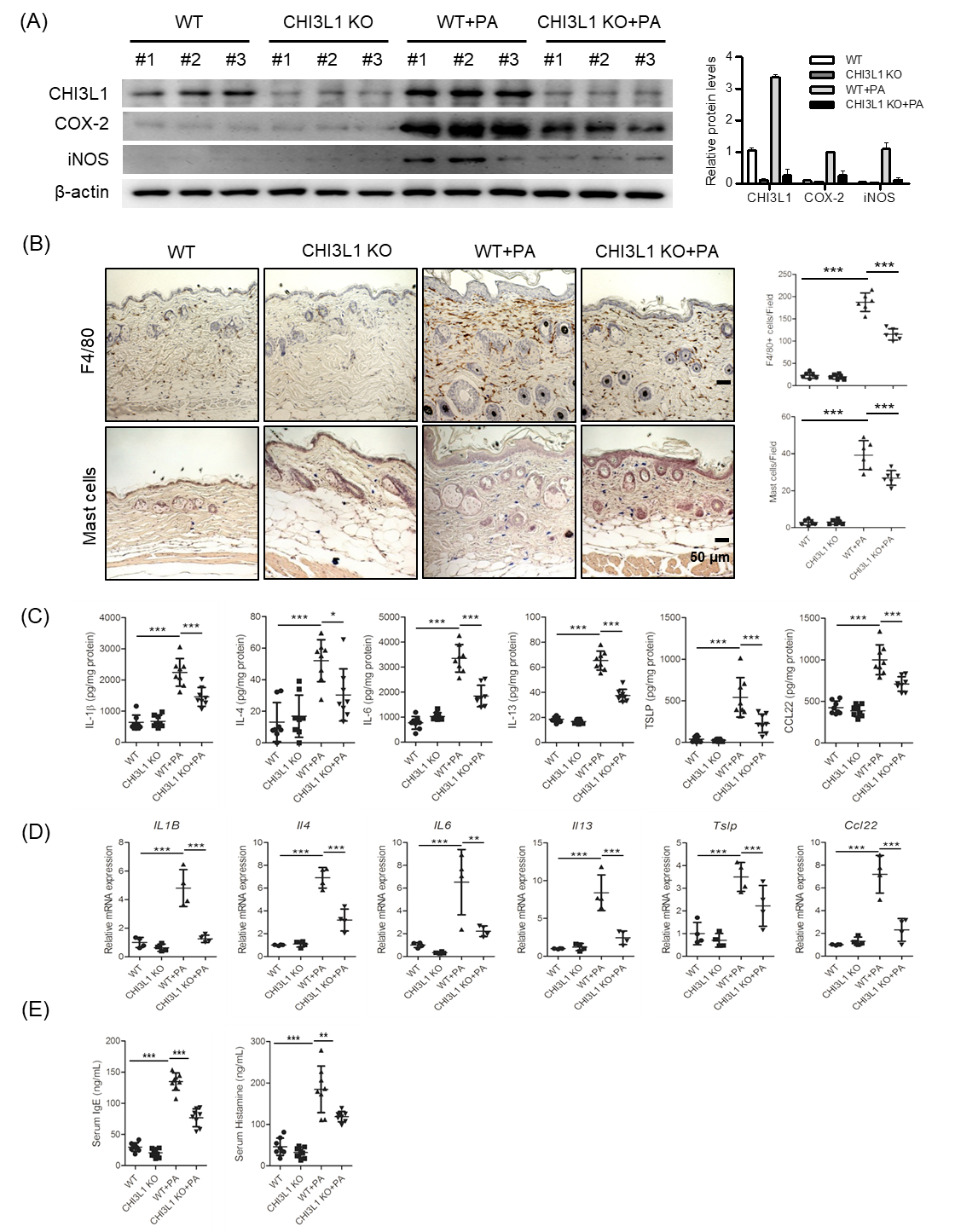
**

**Supplementary Figure S2. CHI3L1 KO suppresses expression of inflammation markers and inflammatory cell infiltration in PA-induced mouse model.** WT and CHI3L1 KO mice were treated with 5% PA for 4 weeks. (A) Expression of inflammation markers, COX-2 and iNOS, in PA-induced skin tissues of WT and CHI3L1 KO mice. (B) Representative immunohistochemistry images showing F4/80 (macrophage) and toluidine blue (mast cell) stained cells in PA-induced skin tissue sections. Scale bar, 50 μm. Bar graphs indicate stained cells quantified by counting the number of positive cells in the field. *n* = 6. (C) Levles of AD-related inflammatory cytokines and chemokines, IL-1β, IL-4, IL-6, IL-13, TSLP and CCL22, in skin tissues. *n*=8. (D) The mRNA expression of AD-related inflammatory cytokines and chemokines, *Il1B, Il4, Il6, Il13, Tslp* and *Ccl22*, in skin tissues. *n*=4. (E) Serum IgE and histamine levels in PA-induced mice. *n*=8. Data are expressed as the mean ± S.D. **p*<0.05, ***p*<0.01 and ****p*<0.001.

**
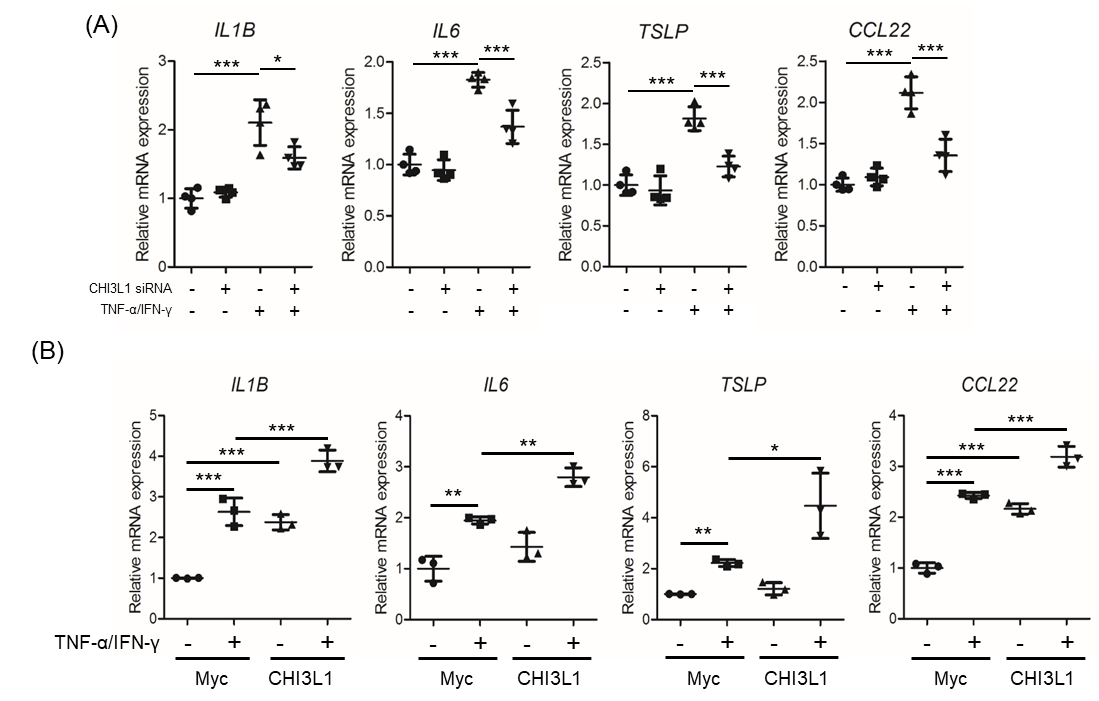
**

**Supplementary Figure S3. CHI3L1 regulates skin inflammatory cytokines and chemokines.** (A) HaCaT cells were transfected with CHI3L1 siRNA (20 nM). After 24 h, the cells were treated with TNF-α/IFN-γ (20 ng/ml). After 24 h, the cells were treated with TNF-α/IFN-γ for 4 h. mRNA expression of *IL1B*, *IL6*, *TSLP*, and *CCL22* was analyzed using qPCR. *n*=4. (B) HaCaT cells were transfected with CHI3L1 plasmid vector. After 24 h, the cells were treated with TNF-α/IFN-γ for 4 h. mRNA expression of *IL1B*, *IL-6*, *TSLP*, and *CCL22* was analyzed using qPCR. *n* = 3. Data are expressed as the mean ± S.D. **p*<0.05, ***p*<0.01 and ****p*<0.001.

**
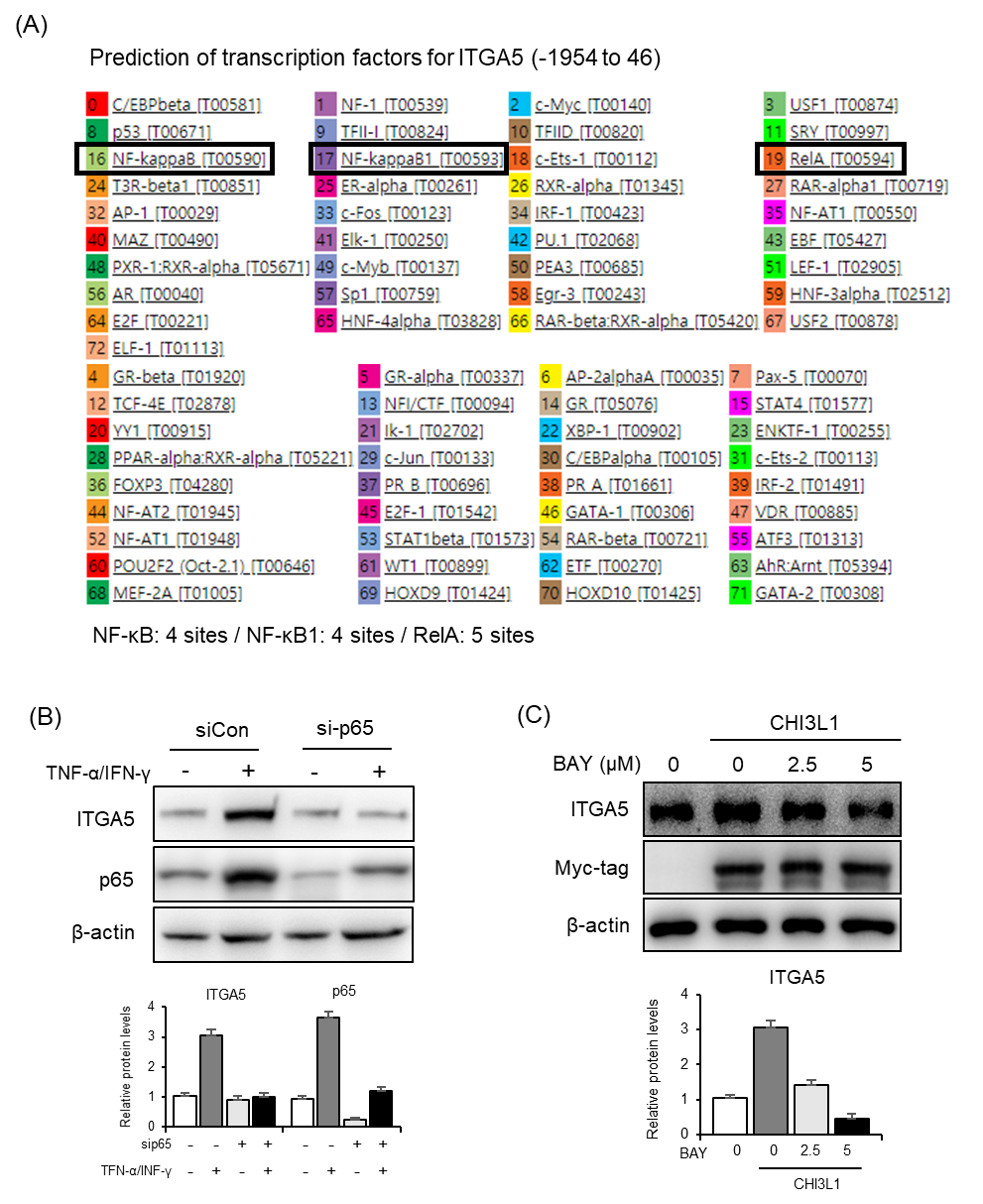
**

**Supplementary Figure S4. ITGA5 is regulated by NF-κB signaling.** (A) Prediction of transcription factor binding sites at ITGA5 promotor region. (B) HaCaT cells were transfected with human p65 siRNA (20 nM). After 24 h, the cells were treated with TNF-α and IFN-γ (20 ng/mL) for 4 h. Expression of ITGA5 was analyzed by Western blot analysis. (C) HaCaT cells were transfected with CHI3L1 plasmid vector for 24 h. Cells were treated with BAY 11-7082 (BAY; NF-κB inhibitor). Expression of ITGA5 was analyzed by Western blot analysis. Data are expressed as the mean ± S.D.

**
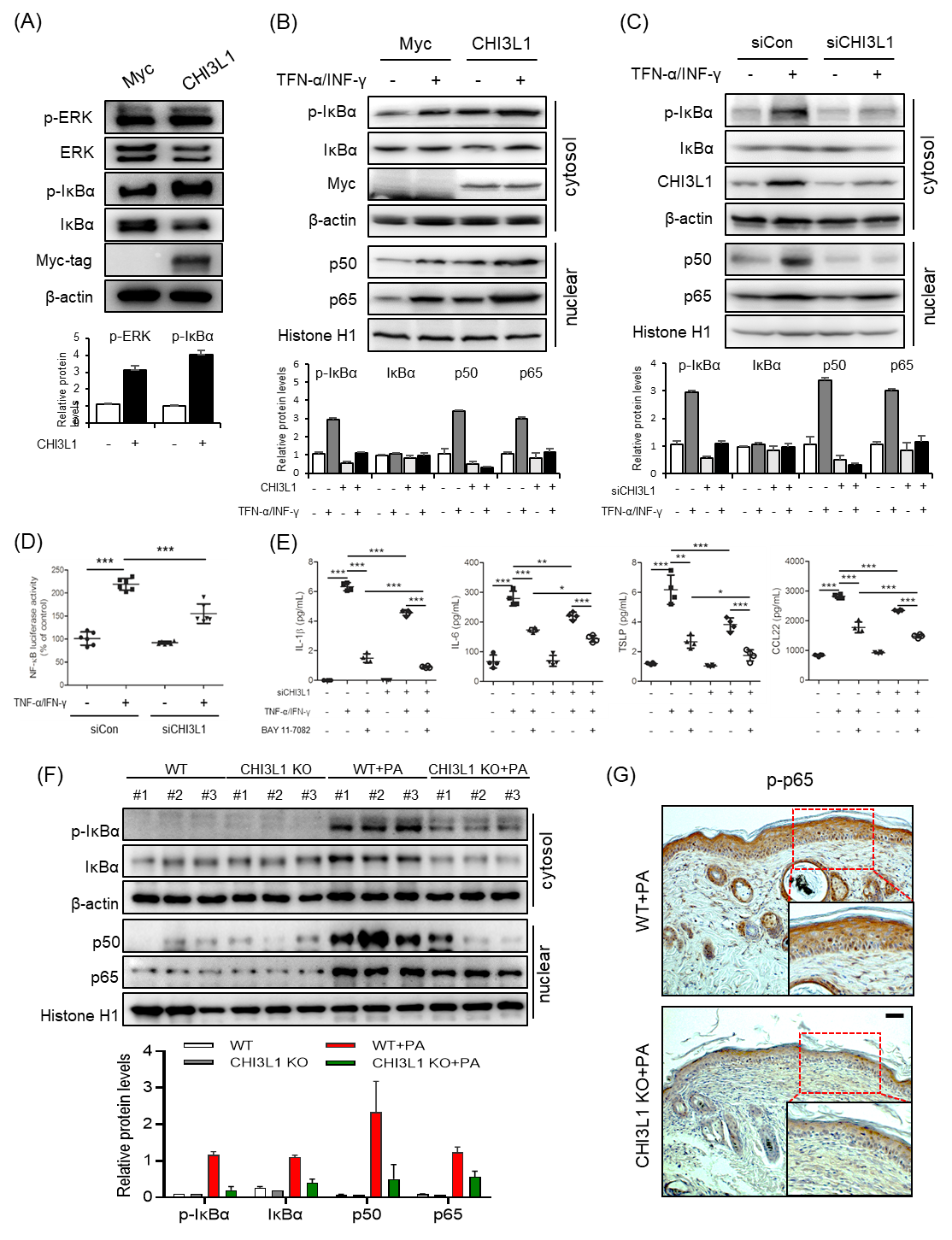
**

**Supplementary Figure S5. CHI3L1 controls NF-κB activation.** (A) HaCaT cells were transfected with CHI3L1 plasmid vector for 24 h. Expression of phosphorylated ERK (p-ERK) and phosphorylated IκBα (p-IκBα) determined by Western blot analysis. (B) HaCaT cells were transfected with CHI3L1 plasmid vector. After 24 h, the cells were treated with or without TNF-α/IFN-γ for 4 h. Expression of IκBα in cytosolic fractions and nuclear translocation of p50 and p65 in nuclear fractions determined in Western blot analysis. (C and D) HaCaT cells were transfected with CHI3L1 siRNA (20 nM). After 24 h, the cells were treated with TNF-α/IFN-γ (20 ng/ml) for 24 h. Expression of phosphorylated IκBα in the cytosolic fractions and nuclear translocation of p50 and p65 in nuclear fractions determined in western blot analysis (C). NF-κB transcriptional activity was measured in a luciferase assay using transfection of luciferase-expressing NF-κB plasmid vector (D). *n* = 6. (E) HaCaT cells were transfected with human CHI3L1 siRNA (20 nM). After 24 h, the cells were treated with TNF-α/IFN-γ with BAY 11-7082 (2.5 μM; NF-κB inhibitor) for 24 h. The levels of pro-inflammatory mediators IL-1β, IL-6, TSLP, and CCL22 were analyzed using ELISA. *n* = 4. (F and G) WT and CHI3L1-KO mice were treated with 5% PA for 4 weeks. Expression of phosphorylated IκBα in cytosolic fractions and nuclear translocation of p50 and p65 in nuclear fractions in PA-induced skin tissues by western blot analysis (F). Expression of phosphorylated p65 (p-p65) in PA-induced skin tissues by IHC analysis (G). The immunohistological images are representative of each group of PA-induced skin tissues. Scale bar, 50 μm. Data are expressed as the mean ± S.D. ***p* < 0.01 and ****p* < 0.001.

**
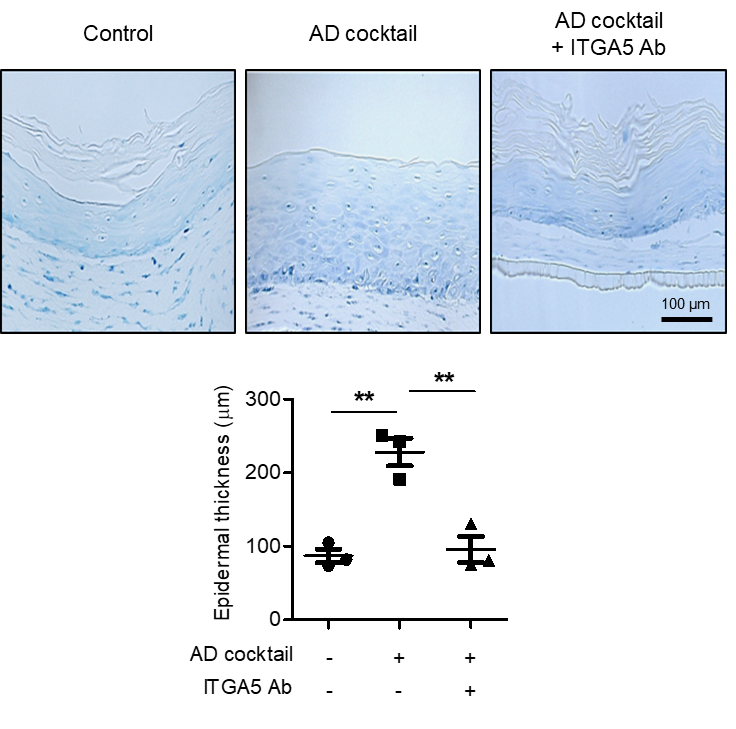
**

**Supplementary Figure S6. Depletion of ITGA5 reduces epidermal thickness in AD-like RHS model.** RHS model cells were cultured with AD cocktail with/without ITGA5 antibody (1 μg/mL) for 6 days. Histological changes in AD-RHS model. Bar graph presents epidermal thickness. *n* = 3. Scale bar, 100 μm. Data are expressed as the mean ± S.D. **p* < 0.05 and ***p* < 0.01.

**
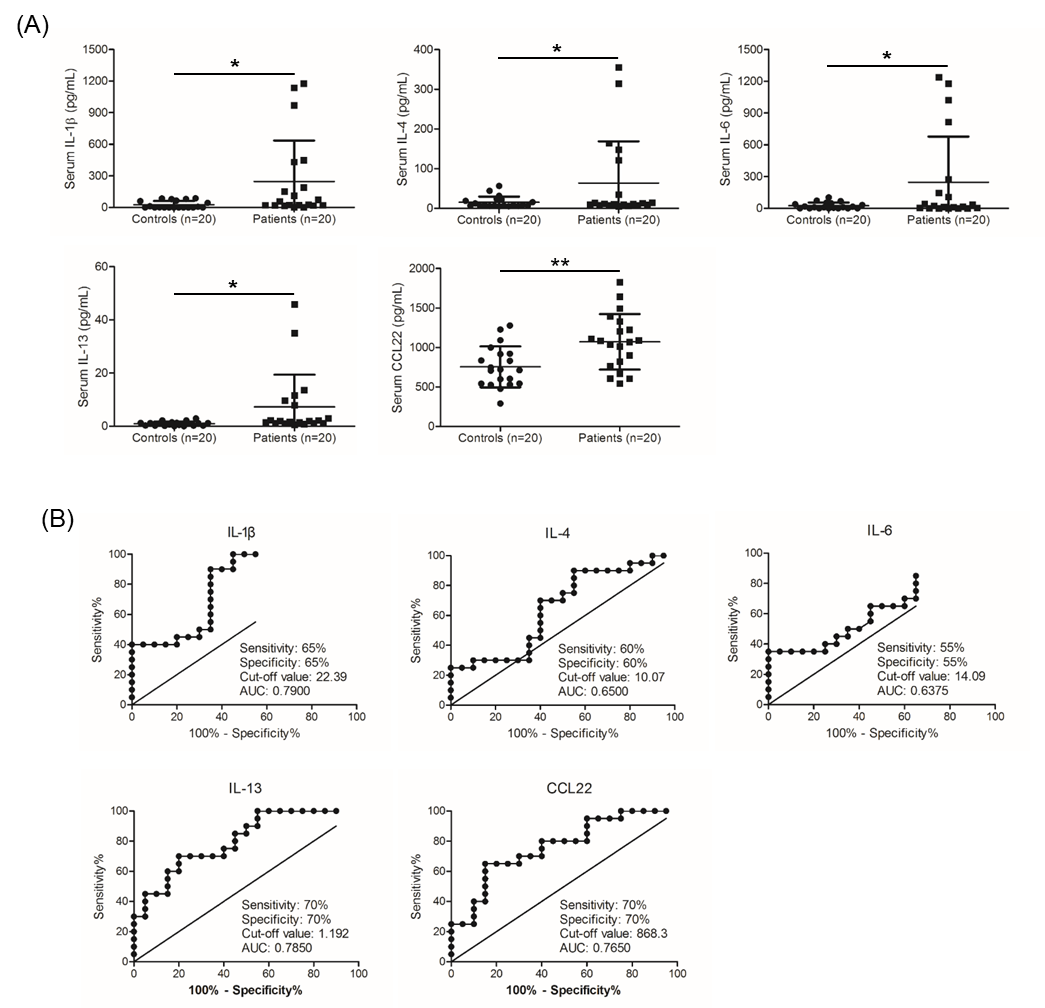
**

**Supplemental Figure S7. Serum analysis of AD-related biomarkers in patients with AD.** (A and B) Serum levels (A) and ROC curves (B) of IL-1β, IL-4, IL-6, IL-13, and CCL22 in patients with AD and healthy controls. *n* = 20. Data are expressed as the mean ± S.D. **p* < 0.05 and ***p* < 0.01.
